# Experimental venous thrombus resolution is driven by IL-6 mediated monocyte actions

**DOI:** 10.1038/s41598-023-30149-2

**Published:** 2023-02-24

**Authors:** Andrea T. Obi, Sriganesh B. Sharma, Megan A. Elfline, Catherine E. Luke, Abigail R. Dowling, Qing Cai, Andrew S. Kimball, Mike Hollinstat, Livia Stanger, Bethany B. Moore, Farouc A. Jaffer, Peter K. Henke

**Affiliations:** 1 Conrad Jobst Vascular Research Laboratories, University of Michigan Medical School, Ann Arbor, USA; 2 Section of Vascular Surgery, University of Alabama Division of Vascular Surgery, University of Michigan Medical School, Ann Arbor, USA; 3 Department of Pharmacology, University of Michigan Medical School, Ann Arbor, USA; 4 Department of Microbiology and Immunology, University of Michigan Medical School, Ann Arbor, USA; 5 Cardiovascular Research Center, Cardiology Division, Department of Medicine, University of Michigan Medical School, Ann Arbor, USA; 6 Section of Cardiology, Massachusetts General Hospital, Harvard Medical School, Boston, USA; 7 University of Michigan Health System, 1500 E. Medical Center Drive, Cardiovascular Center - 5463, Ann Arbor, MI 48109-5867 USA

**Keywords:** Medical research, Experimental models of disease

## Abstract

Deep venous thrombosis and residual thrombus burden correlates with circulating IL-6 levels in humans. To investigate the cellular source and role of IL-6 in thrombus resolution, Wild type C57BL/6J (WT), and IL-6^−/−^ mice underwent induction of VT via inferior vena cava (IVC) stenosis or stasis. Vein wall (VW) and thrombus were analyzed by western blot, immunohistochemistry, and flow cytometry. Adoptive transfer of WT bone marrow derived monocytes was performed into IL6^-/-^ mice to assess for rescue. Cultured BMDMs from WT and IL-6^−/−^ mice underwent quantitative real time PCR and immunoblotting for fibrinolytic factors and matrix metalloproteinase activity. No differences in baseline coagulation function or platelet function were found between WT and IL-6^−/−^ mice. VW and thrombus IL-6 and IL-6 leukocyte-specific receptor CD126 were elevated in a time-dependent fashion in both VT models. Ly6C^lo^ Mo/MØ were the predominant leukocyte source of IL-6. IL-6^−/−^ mice demonstrated larger, non-resolving stasis thrombi with less neovascularization, despite a similar number of monocytes/macrophages (Mo/MØ). Adoptive transfer of WT BMDM into IL-6^−/−^ mice undergoing stasis VT resulted in phenotype rescue. Human specimens of endophlebectomized tissue showed co-staining of Monocyte and IL-6 receptor. Thrombosis matrix analysis revealed significantly increased thrombus fibronectin and collagen in IL-6^−/−^ mice. MMP9 activity in vitro depended on endogenous IL-6 expression in Mo/MØ, and IL-6^−/−^ mice exhibited stunted matrix metalloproteinase activity. Lack of IL-6 signaling impairs thrombus resolution potentially via dysregulation of MMP-9 leading to impaired thrombus recanalization and resolution. Restoring or augmenting monocyte-mediated IL-6 signaling in IL-6 deficient or normal subjects, respectively, may represent a non-anticoagulant target to improve thrombus resolution.

## Introduction

In experimental models of venous thrombosis (VT), leukocytes, chemokines, and pro-inflammatory cytokines determine the time course and phenotype of thrombus resolution and vein wall healing^1–5^. This process resembles local wound healing, with early influx of neutrophils (PMN), followed by monocytes/macrophages (Mo/MΦ)^1,6,7^. VT resolution is highly dependent on local monocyte infiltration: exogenous administration of chemokines CCL2 (MCP-1) or IL-8 accelerates resolution, whereas genetic deletion of CCR2 or CXCR2 impairs the process^3,6,8^. Harnessing the immune system in VT thus represents a potentially promising, non-anticoagulant based strategy to treat DVT and prevent morbid sequelae such as post thrombotic syndrome (PTS), as no current direct medical therapy exists^9^.

Recent human studies suggest that circulating IL-6 is a marker of incident PTS and DVT burden^10,11^. Post thrombotic circulating IL-6 levels correlate with venous outflow resistance, a measure of venous obstruction and contributing factor in PTS pathophysiology^12^. IL-6 is a common acute phase pro-inflammatory cytokine released from the liver and leukocytes with multiple end-organ effects^13,14^. IL-6 signaling occurs via direct receptor binding on leukocytes (CD126) or more ubiquitously via trans-signaling via soluble IL-6Rα (sIL-6Rα)^14,15^. The IL-6/sIL-6Rα binds to receptor gp130 activating downstream JAK-STAT3 pathways. IL-6 signaling is important for regulating transition from PMN to Mo/MØ-driven inflammatory process and fibrosis^16^, as well as leukocyte recruitment^15^. Importantly, IL-6 may play an active role in VT resolution, in part by regulating fibrinolytic and MMP activity in Mo/MØ through spatial and temporal regulation of enzyme expression^17,18^.

Given the relationship of circulating IL-6 to persistent human DVT and an established role for leukocytes in thromboinflammation, in this study we hypothesized that: IL-6 would be locally elevated in experimental venous thrombosis in both the thrombus and vein wall; that thrombus leukocytes would be a major source of thrombus IL-6; and that IL-6 would play a critical role in thrombus resolution.

## Methods

### Animals

C57BL/6J (male, 20–25 gm) and IL-6^−/−^ mice (B6.129S2-il6tm1kopf/J PRID:IMSR_JAX:002650 Strain #002650), all 8–12 weeks of age, were obtained from the Jackson Laboratory (Bar Harbor, ME) and were used for all studies. Male mice were chosen because the risk of PTS and recurrent DVT is highest in men^19,20^. Previous data suggests that the parameters of thrombus weight and histology do not differ between male and female mice at the timepoints studied (Luke and Henke, unpublished data). Furthermore, the thrombosis models require ligation of side branches draining into the IVC and this commonly includes the right uterine vein in females resulting in reproductive organ necrosis and significant morbidity, increasing the animal number required^21^. Therefore balancing the responsible and humane use of animals against benefit of knowledge gained, we determined a priori that only male mice would be used. The animals were housed within SPF facilities in the Unit for Laboratory Animal Medicine at the University of Michigan North Campus Research Complex. The Institutional Care and Use Committee (IACUC) approved and surveyed all protocols conducted herein (protocol # PRO00010231). The study design and execution and reporting is in accordance with the ARRIVE guidelines. The animals were cared for in accordance with “Guide for the Care and Use of Laboratory animals’ published by the NIH (Publication 85–23, National Academy Press, Washington, DC).

### Stasis model of venous thrombosis (VT)

VT was created by inducing complete blood stasis via infrarenal IVC ligation as previously described^9,15,16^. Briefly, mice were initially sedated in an induction chamber with 4% isoflurane and oxygen, and then anesthetized by 2% inhaled isoflurane via nose cone. A midline laparotomy was performed, viscera were rotated laterally to expose the IVC and the venous side branches and dorsal branches were cauterized or ligated. The infrarenal IVC was ligated with a 7–0 prolene suture (Ethicon Inc., Somerville NJ) to generate stasis thrombosis. IVC, thrombi and plasma were harvested at 4, 8 and 21 days following IVC ligation^22^. The IVC and thrombus of each mouse was first measured (cm) and weighed (grams) to quantify size. The specimens were then separated and snap frozen in liquid nitrogen. At the final time point (21 days), the vein wall and thrombus are inseparable due to fibrotic changes, and hence, the tissue must be analyzed together.

### Stenosis model of VT

In the stenosis model, under the same conditions as the stasis model, the infrarenal IVC is similarly exposed and side branches ligated, however, a 30-gauge needle is placed along the ventral surface of the IVC and a 7–0 prolene suture is cinched around both the needle and vein. Following this partial ligation, the needle is removed, and the result produces a consistent ~ 95% narrowing of the IVC^23^. As previously demonstrated, thrombosis is less consistently achieved with this model, however, the hemodynamics are more consistent with clinical partially occlusive thrombosis^24^. Tissue specimens are processed in an identical fashion as the stasis model.

### Sham surgery animals

Control mice were housed and treated under the same conditions as the stasis and stenosis mice; however, a sham surgery was performed. Specifically, control mice underwent laparotomy with retroperitoneal dissection and passage of surgical instrument under the IVC but without venous ligation. Similar to stasis and stenosis mice, tissue specimens are collected and processed as described above.

### Platelet and coagulation assessment

#### Mouse platelet isolation^25^

Wild-type and IL6^−/−^ mice were anesthetized with isolflurane anesthesia and whole blood was collected through cardiac puncture at a 1:10 dilution in 3.2% sodium citrate. 100 µL of blood was used for PT and aPTT test using the VETSCAN VSpro Coagulation Analyzer, Zoetis, Parsnippany, NJ. Platelet rich plasma (PRP) was isolated from the remaining citrated whole blood though serial centrifugation at 200×*g* for 5 min. The PRP was treated with acid citrate dextrose (2.5% sodium citrate tribasic, 1.5% citric acid, and 2.0% d-glucose) and apyrase (0.1 U/mL), and then centrifuged for 8 min at 2000×*g* to pellet the platelets. Washed platelets were resuspended in Tyrode's buffer at 3 × 108 platelets/mL to be used in flow cytometry. Platelets were recalcified to a final concentration of 1 mM with CaCl_2_ before stimulation in flow cytometry studies.

#### Flow cytometry

Washed platelets from wild-type and IL6^-/-^ mice were stimulated with either 0.5 nM thrombin or 50 ng/mL convulxin (Cayman Chemicals) and stained with a FITC-conjugated antibody specific for the active conformation of α2bβ3 (PAC1, BD Pharmingen) and an anti-CD62P-APC-Cy7 antibody (BioLegend, San Diego, CA). Platelets were incubated for 10 min at 37 °C and fixed with 2% paraformaldehyde. Surface expression of activation markers was analyzed with a CytoFLEX flow cytometer (Beckman Coulter, Brea, CA).

Mouse blood at base line was run for PT, aPTT using VETSCAN VSpro Coagulation Analyzer, Zoetis (formerly Abaxis), Parsippany, New Jersey.

### Western blots

RIPA buffer (ThermoScientific, Rockford, IL, USA) and complete ULTRA Mini Tablets (Roche, Mannheim, Germany) was used to isolate protein from thrombus and IVC segments and cultured BMDMs or immortalized murine macrophages (RAW264.7 cells). For MMP9 and urokinase analysis, BMDMs and RAW264.7 cells were treated with either IL6 (20 ng/mL) or Brefeldin A (protein transport inhibitor; 500 ng/mL for 3 h). Protein concentration of these lysates was determined using the BCA assay (Thermo Fisher). Protein separation was then achieved by electrophoresis using either NuPAGE 4–12% Bis Tris gels (Invitrogen, Carlsbad, CA) or fixed on percentage SDS polyacrylamide gels. Proteins were then transferred onto PVDF membranes (Millipore, Billerica, MA, USA) and probed with the indicated primary antibodies (and antibody concentration): IL-6 (@1:1000, NB600-1131, Novus Biologicals, Centennial CO), gp130 (@1:1000, bs-1459R, Bioss Biocompare, Woburn, MA), CD126 (@1:1000, GTX37399, GeneTex, Irvine, CA), fibronectin (@1:1000, ab2413, Abcam, Cambridge, MA), MMP9 (@ 1:1000, ab38898, Abcam, Cambridge, MA), integrin α V, (@1:1000, ab179475, Abcam, Cambridge, MA), urokinase (@1:1000, ab24121, Abcam, Cambridge, MA) Bound antibodies were subsequently probed with the indicated secondary antibodies: Goat Anti-Rabbit IgG H&L (Cy2 ®) preadsorbed (@ 1:1,000, ab6940, Abcam, Cambridge, MA), Goat Anti-Mouse IgG H&L (Cy3 ®) preadsorbed (@1:1,000, ab97035, Abcam, Cambridge, MA). Immunoreactive bands were detected using SuperSignal West Pico Chemiluminescent Substrate (REF34577, ThermoScientific, Rockford, IL) and GE Amersham 600 fluorescent imager. Optical densities were normalized to β actin (1:25,000, Santa Cruz Biotechnology, Dallas, Tx) and total protein loading as assayed by colloidal coomassie staining of PVDF membranes and immunoblot band quantitation was performed using Image J software.

### Histology

Retroperitoneal contents including the IVC, associated thrombus, and aorta were dissected from each mouse en bloc, fixed in formalin, embedded in paraffin, cut into 5 mm sections and mounted on glass microscope slides as previously described. Slides were stained for H&E for morphological evaluation. Antigens stained for included vWF (1:500, Abcam, Cambridge, MA) CCR2 (1:800, abNBP2-67700, Novus Biologicals, Centential CO), CXCR2 (1:500, ab19538-1-AP, Preprotech, Rosemont, IL), and CD31 (1:100, abAb28364, Abcam, Cambridge, MA). For immunohistochemical staining, slides were deparaffinized with xylene and ethanol, rehydrated to water and processed for antigen retrieval. Nonspecific binding was blocked with normal serum and sections were then immunostained with primary antibody. Rabbit IgG was used as negative control (same dilutions as primaries, ab172730, Abcam, Cambridge, MA). A horseradish peroxidase-conjugated secondary antibody (Anti-Rabbit IgG) was then applied followed by DAB substrate according to the manufacturer’s instructions (Vector Laboratories Inc., Burlingame, California). The slides were counterstained with hematoxylin, rinsed, allowed to dry and cover slipped with Acrytol Mounting Medium (Electron Microscopy Science, Hatfield, PA). In a blinded fashion, thrombus neovascular channels and cell counts were tabulated from five representative high power fields, from 3 sections in the mid-VT per mouse^3,26^.

Martius Scarlet Blue (MSB) staining was used to evaluate thrombus collagen and fibrin. Tissue slides were deparaffinized through xylene and gradient ethanol (EtOH), and rehydrated to distilled water (diH2O). Postfix was performed in Bouin Fixative (Newcomer Supply, Inc., Middletown, WI) at 60 degrees for 1 h, slides were cooled for 30 min and washed in diH2O for 15 min. to remove traces of picric acid. Nuclei were stained by Weigert’s Hematoxylin (Fisher Chemical, Fair Lawn, NJ) for 10 min. and washed in diH2O for 5 min. Slides were rinsed and dehydrated in 16 dips of diH_2_O followed by 16 dips in 95% EtOH. Red blood cells were stained with Martius Yellow (Fisher Chemical, Fair Lawn, NJ) for 2 min. Slides were washed in 16 dips of 2 consecutive sets of clean diH2O then submerged in Methyl Blue (Sigma Aldrich, St. Louis, MO) for Collagen for 20 min, then washed in 16 dips of 2 consecutive sets of clean diH2O, then submerged in Crystal Scarlett (Crystal Ponceau 6R, MP Biomedicals LLC, Solon, OH) for fibrin for 5 min. Slides were washed, rapidly dehydrated and cleared with 16 dips of 2 consecutive sets of clean diH2O, 8 dips in 100% EtOH, 6 dips in Xylene and then cover slipped with Acrytol (Electron Microscopy Science, Hatfield, PA) and allowed to dry overnight. Whenever possible, stain compounds were certified by Biological Stain Commission, Inc. Images were taken at 100 × using a Nikon Eclipse E400 brightfield microscope with Digital Sight camera (DS-Ri1) and controller (DS-U4). Red, blue and yellow staining was analyzed with Image J Macros software.

#### Human studies

Slides from human chronic iliofemoral DVT, from patients undergoing endophlebectomy were deparaffinized and rehydrated sequentially with xylene, 100%, then 50% ethanol, and water. Antigen retrieval was performed using heat-mediated Sodium Citrate buffer (10 mM NaCL solution, pH 6.0 at 95 degrees for 10 min then allowed to cool for 20 min). Nonspecific binding was blocked with normal serum and sections were then immunostained sequentially with gp130 (@1:100, BS-1459R, bs-1459R,Bioss Biocompare, Woburn, MA), Alexa Fluro 594 (@1:500, A-21442, ThermoFisher, Waltham, MA), CD68 (@1:200, MS-397-P, ThermoFisher, Waltham, MA), and Alexa Fluro 488(@1:500, A-11029, ThermoFisher, Waltham, MA), then counterstained and cover slipped with ProLong Gold antifade reagent with DAPI (REF P36931, Invitrogen by Thermo Fisher Scientific, Waltham, MA), Photos were taken using a Nikon B400 microscope with Spot camera. The study protocol has been previously published, reviewed and approved by the governing institutional review board^27^.

### Cell extraction for flow cytometry

Thrombus and IVC samples were immediately placed in RPMI and sharply minced as previously described^29^. Briefly, enzymatic digestion was performed with DNase I (Sigma-Aldrich) and Liberase (Sigma-Aldrich) at 30 °C for 30 min. RPMI and fetal bovine serum were used to stop the reaction. Samples were plunged 30 times with a 3 mL syringe, then filtered through a 100 µM filter. The cells were washed in PBS and resuspended.

### Flow cytometry

Single cell suspensions were stained in 1.5 mL centrifuge tubes for flow staining. Cells were first stained with a Fixable LIVE/DEAD viability dye (Molecular Probes by Life Technologies; Ref. L34959; 1:2000 dilution). Following viability staining, cells were washed in PBS and resuspended in flow buffer (PBS, FBS, NaN3, HEPES). FcR-receptors were then blocked with anti-CD16/32 (BioXCell, Cat. CUS-HB-197, 1:400 dilution) for 10 min. Monoclonal antibodies for surface staining included: Anti-CD3 (Biolegend, Cat. 100304, 1:400 dilution), Anti-CD19 (Biolegend, Cat. 115504, 1:400 dilution), Anti-Ter-119 (Biolegend, Cat. 116204, 1:400 dilution), Anti-NK1.1 (Cat. 108704, 1:400 dilution), Anti-Ly6G (Biolegend, Cat. 127604, 1:400 dilution), Anti-CD11b (Biolegend, Cat. 101230, 1:400 dilution), Anti-Ly6C (Biolegend, Cat. 128035, 1:400 dilution) and Anti-F4/80 (Biolegend, Cat. 123121, 1:400 dilution) as previously described^28–33^. Following surface staining, cells were washed, and biotinylated antibodies were labeled with streptavidin-fluorophore (Biolegend, Cat. 405208, 1:1000 dilution). Stained samples were then washed twice in flow buffer and acquired on a 3-Laser Novocyte Flow Cytometer (Acea Biosciences) or FACS sorted on a FACS Aria III Flow Sorter. FACsDiva Software (BD Biosciences) was used for flow sorting, FlowJo version 10.0 (Tree Star) was used for flow cytometric analysis. Back gating of cell populations was performed to verify gating and purity.

### Bone marrow-derived macrophage culture and adoptive transfer

Bone marrow-derived macrophages (BMDMs) from WT and IL6^−/−^ mice were generated as previously described^30^. The purity of cells was over 95% (Supplemental Fig. 1)^34^. Where indicated, BMDMs were treated with recombinant murine IL6 (20 ng/mL final concentration, R&D systems, Minneapolis, MN) on post-harvest day 6. The IL-6 dose was chosen based on previous published data^35^ and confirmed via dose response curve. Immortalized murine macrophages (RAW264.7 cells) were obtained from the American Type Culture Collection (ATCC; Manassas, VA) and were maintained in DMEM supplemented with 10% FBS, l-glutamine, and penicillin/streptomycin.

Bone marrow cells were collected by flushing femurs and tibias of TdTomato expressing mice (8–10 weeks old) with RPMI 1640 (Lonza). On day 6 following harvest, unpolarized BMDMs were washed with cold PBS, and resuspended in L-15 media for intravenous injection to the recipient mice (10–12 weeks old) with 5 × 10^6^ cells injected intravenously on post-VT induction day 4^33^.

### RNA isolation, reverse transcription, and real time-PCR detection

Total RNA was isolated from the indicated cells using Qiagen RNeasy mini kits (Thermo Fisher) or Nucleospin RNA kits (Takara Bio USA, Mountain View, CA). RNA was reverse transcribed using superscript III (Invitrogen) reverse transcriptase. mRNA transcripts were analyzed using Power Sybr Green qPCR mastermix (Invitrogen) with the following specific primers: (Supplemental Table 1). Reactions were normalized to *Rpl38*. PCR reactions were performed using the Applied Biosystems 7900ht Fast PCR system (Thermo Fisher). mRNA levels were determined by the threshold cycle (ΔΔCT) method. Three independent experiments were performed in triplicate fashion.

### In vitro MMP activity assay

Analysis of total MMP activity of indicated BMDM cells was performed using a commercially available fluorescence based MMP activity assay kit (ab112146, Abcam, Cambridge, MA) per the manufacturers’ instruction. Briefly, the indicated cells were lysed in ice cold 0.1% (V/V) Triton X-100 in PBS at a concentration of 40 million cells per 1 mL of lysis buffer. 50 µL of lysates per sample were pipetted in duplicate fashion with the provided fluorescent MMP substrate, and incubated at 37 °C prior to fluorimetry at 490/525 nm wavelength.

### Statistical analysis

All data are represented as mean ± standard deviation (SD). The D’Agostino-Pearson test was used to determine normality. Parametric or non-parametric tests were used accordingly. Parametric analysis to evaluate differences between experimental and control groups included unpaired t-test with Welch’s correction and one-way ANOVA followed by Dunnett’s post hoc analysis. Nonparametric analysis included Mann–Whitney *U* test or Kruskal–Wallis test. Sigmoidal 4-point logistic regression curves and half-maximal activity concentrations (denoted EC_50_) were generated for the relative fluorescence output yielded from the MMP activity assay. A p < 0.05 was considered significant. Graph Pad Prism version 6.01; (Graph Pad Software, San Diego, CA) was utilized for statistical analysis.

## Results

### Murine local post-thrombotic inflammation is characterized by a time-dependent increase in IL-6 and leukocyte specific IL-6 receptor CD126

**Figure 1:**
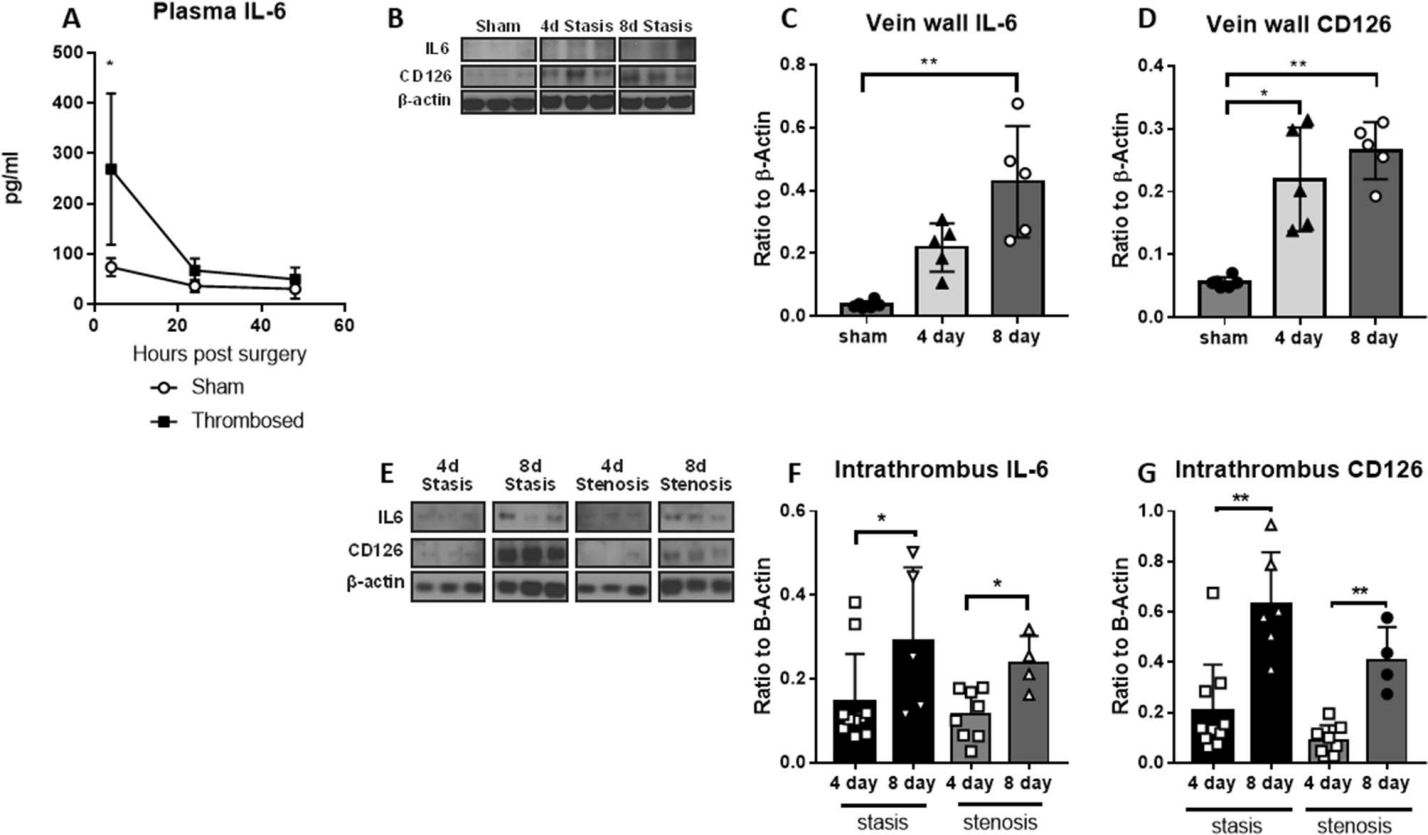
IL-6 and IL-6 receptors are elevated in murine and human post thrombotic tissue. A**.** Plasma IL-6 levels drawn following sham surgery with retroperitoneal dissection or surgery with IVC ligation in WT mice resulting in stasis thrombosis (n = 5 per group). Following sham surgery or stasis thrombosis, vein wall lysate protein levels (**B**) of IL-6 (**C**) and CD126 (**D**) measured by Western blotting (n = 4–10/group). Gel blots cropped from different parts of the gel are presented, with white line separation. Protein levels of intrathrombus (**E**) IL-6 (**F**) and CD126 receptor (**G**) were measured at 4 and 8 days in partially occlusive (stenosis) and completely occlusive thrombi (stasis). All data presented as mean ± standard deviation. Normally distributed data was compared utilizing unpaired student’s test with Welch’s correction and non-normally distributed data compared with Mann–Whitney U test. *p < 0.05, **p < 0.01.

While robust data demonstrates elevated circulating blood levels of IL-6 following acute thrombosis^36,37^, there is a paucity of tissue-level information regarding local IL-6 signaling in DVT resolution. We thus assessed the role of IL-6 in two murine models of acute and chronic VT. Consistent with human DVT^38^, mice showed very early (< 24 h) systemic elevation of circulating IL-6, with a subsequent decline over time (Fig. 1A). Thrombi and vein walls were isolated from mice undergoing either venous stenosis or stasis models at 4 and 8 days. The post thrombotic vein wall and thrombus demonstrated significant increases in IL-6 from day 4 to day 8 post thrombosis (Fig. 1B,C,E,F). A significant increase in thereceptor CD126 was observed in both the vein wall and thrombus at day 8, corresponding with the VT-induced inflammatory cell infiltrate^39^ (Fig. 1B,D,E,G). Ubiquitous non-specific IL-6 receptor gp130 was elevated early in the vein wall and did not demonstrate significant variation within the thrombus over time (data not shown).

### IL-6 is an essential determinant of late thrombus matrix composition, neovascularization and resolution

**Figure 2:**
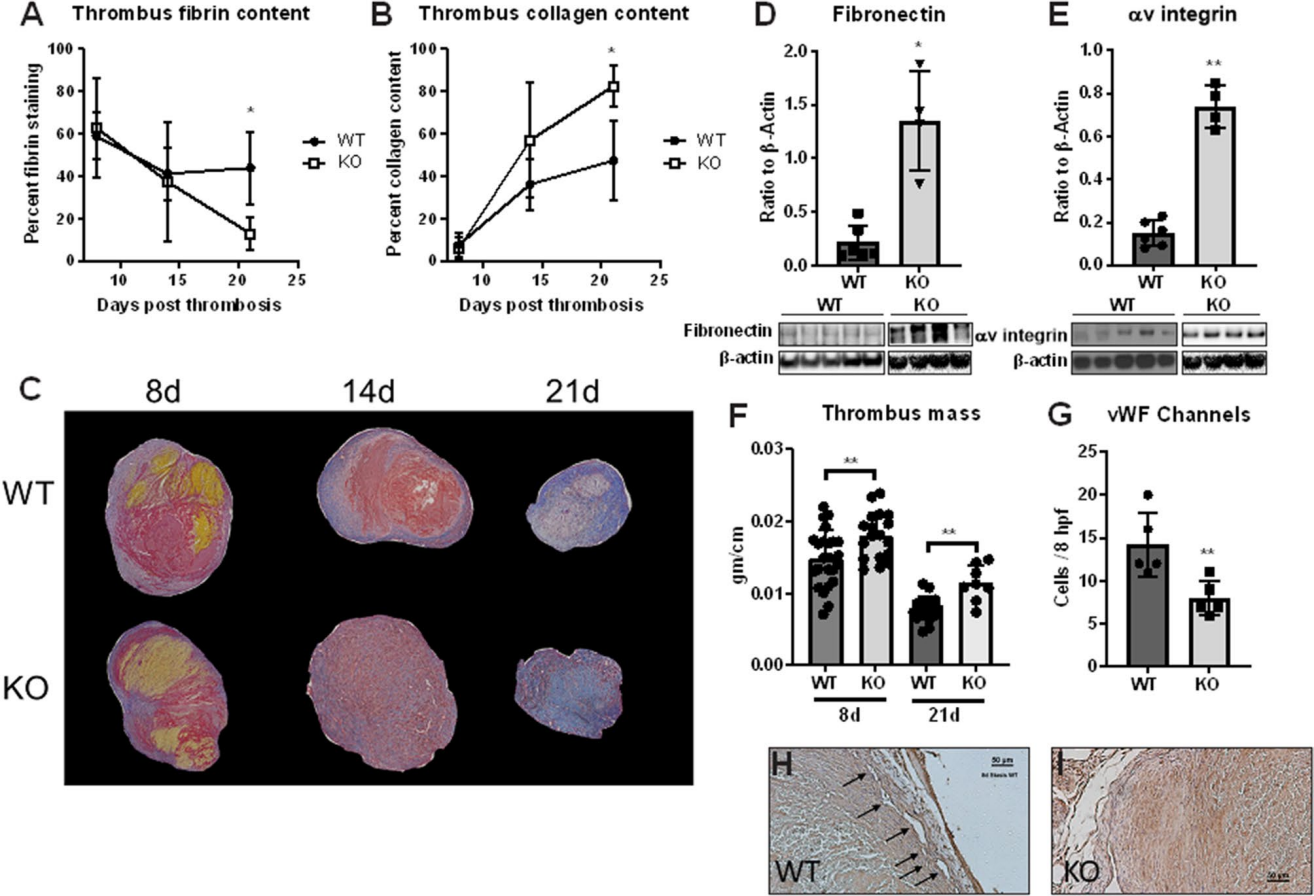
Loss of IL-6 signaling results in impaired thrombus resolution, increased collagen deposition and reduced recanalization. IL-6^−/−^ and WT mice underwent venous thrombosis via IVC ligation and thrombus evaluated for composition at 8, 14 and 21 days. Martius Scarlet and Blue trichrome staining was performed to determine relative fibrin (**A**) and collagen (**B**) content over time (each data point representative of ≥ 3 analyses). Representative mosaic photos of isolated thrombus without adjacent vein wall of WT and IL6^−/−^ shown in (**C**); red staining indicative of fibrin, yellow of erythrocytes and blue of collagen. Total thrombus lysate was evaluated at 8 days by western blotting for glycoprotein fibronectin (**D**) and receptor integrin αv (n = 4–6/group) (**E**) Gel blots cropped from different parts of the gel are presented, with white line separation. Thrombus resolution was quantified via measurements of thrombus weight and length at mid (8 days) and late (21 days) timepoints post thrombosis (**F**). Thrombus recanalization was quantified via presence of positively stained vWF channels within 8 hpf (n = 4–6/group) (**G**). Representative photomicrographs at 40 × of WT (left, arrows mark recanalization channels) and IL-6^−/−^ shown in **H** and** I**. All data presented as mean ± standard deviation. Normally distributed data was compared utilizing unpaired student’s test with Welch’s correction and non-normally distributed data compared with Mann–Whitney *U* test. *p < 0.05, **p < .01.

The process of VT resolution is complex and involves the degradation of thrombus by fibrinolysis, neovascularization with formation of endothelial lined channels within the maturing thrombus, and conversion of the thrombus from a fibrin-rich to a collagen-rich structure^7,40,41^. To determine how IL-6 impacted this process we quantitatively evaluated thrombus composition over time in WT and IL-6 deficient mice (IL-6^−/−^). Global gene deletion of IL-6 was associated with a fibrin-deficient, collagen-rich thrombus during late thrombus resolution (Fig. 2A–C), with no difference in rbc quantity over time (8d ~ 15–16%; 14d 1–1.5%, and 21d 1.1–1.2%, P = NS). Collagen deposition is often preceded by excessive fibronectin deposition^42–46^. We discovered that preceding late collagenous transformation of the thrombus, the structural components fibronectin and binding partner αv integrin were elevated in IL-6^−/−^ compared to WT (Fig. 2D,E). These findings associated with impaired thrombus resolution at mid and late time points in stasis VT (Fig. 2F), and at the late time point in the stenosis VT (Supplemental Fig. 2). Of note, we found no difference in platelet function or coagulation assays between WT and IL-6^−/−^ mice, and the difference in thrombus size was not dependent on mouse weight (Supplemental Figs. 3 and 4). Initial thrombus size during thrombogenesis was not impacted as measured by thrombus size at 2 days (Supplemental Fig. 5). Thrombus morphology was analyzed for recanalization, and we found significantly fewer thrombus neovascular channels in IL-6^−/−^ mice as compared with WT mice (Fig. 2G–I), by both vWF + staining, and CD31 + staining (Supplemental Fig. 6). Lastly, we found no difference in thrombus CXCR2 positive cells, but a strong trend in CCR2+ cells, with greater nunbers in IL-6^−/−^ as compared with WT (Supplemental Fig. 7).

### Ly6C + monocytes/macrophages are the primary myeloid cellular source of IL-6 in the resolving thrombus

**Figure 3:**
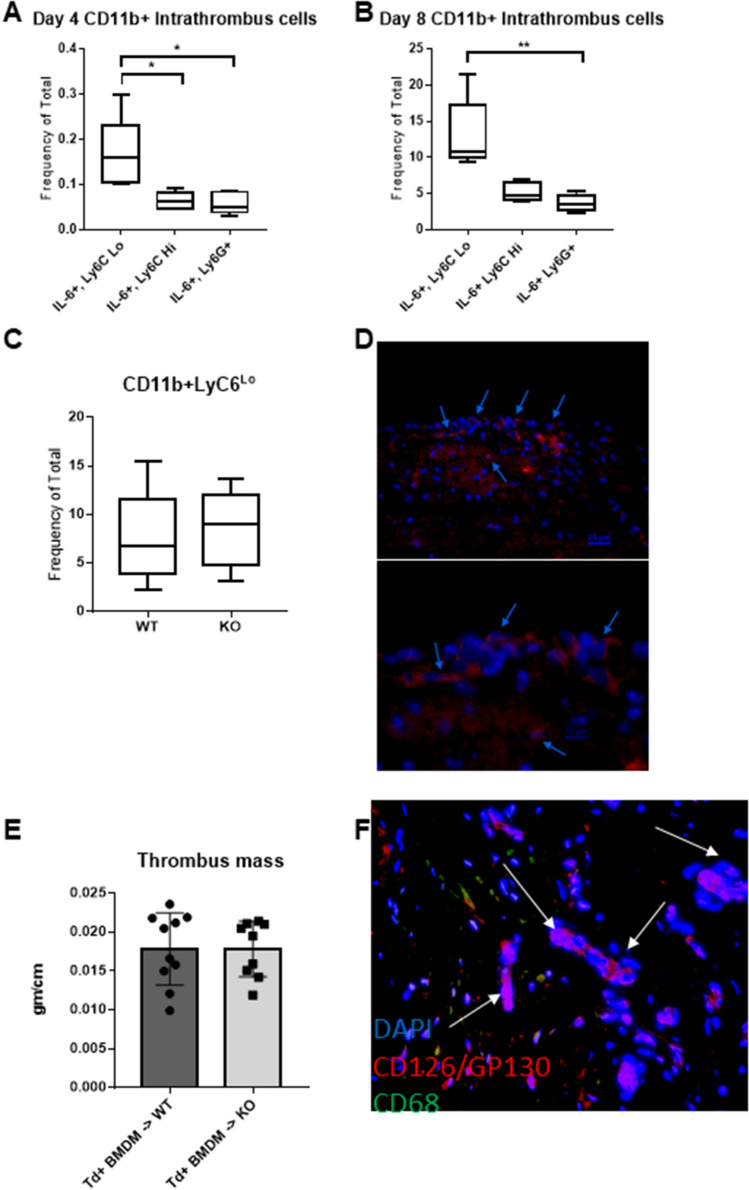
Adoptive transfer of the primary IL-6 + leukocyte within the thrombus restores normal thrombus resolution. At 4 and 8 days following IVC ligation, thrombus was isolated, digested and sorted by flow cytometry to identify leukocyte source of IL-6. The frequency of IL-6 + (CD11b + [CD3-CD19-Ter119-NK1.1-]Ly6C^lo^ and Ly6C^hi^ monocytes and Ly6G + neutrophils within thrombus at day 4 (**A**) and day 8 (**B**). The frequency of Ly6C^lo^ (CD11b + CD3-CD19-Ter119-NK1.1-) cells in WT compared to IL-6^−/−^ mice at 8 days post thrombosis is shown in (**C**). Adoptive transfer of monocytes from mTmG mice was performed into WT and IL-6^−/−^ mice on day 4 post thrombosis. (**D**) Representative immunofluorescence microscopy of thrombus 4 days following adoptive transfer in WT mouse, blue arrows denote Td red monocytes counterstained with DAPI in day 8 thrombus. (**E**) Thrombus mass measured 4 days following adoptive transfer. (**F**) Immunofluorescence microscopy at 100 × of human chronic DVT specimens**.** White arrows denote cells co-staining with DAPI (blue), CD68 (green) and gp130 (red). The image is representative of 3 patient histologic samples. All data presented as mean ± standard deviation. Normally distributed data was compared utilizing unpaired student’s test with Welch’s correction and non-normally distributed data compared with Mann–Whitney *U* test. For comparisons across > 2 groups, Kruskal–Wallis testing was performed *p < 0.05, **p < 0.01.

Given the central role of Mo/MΦ during thrombus resolution and in formation of neovascular channels^3,33^, flow cytometry of the thrombosed vein at 8 days was used to assess IL-6 positive infiltrating cells amongst CD11b^+^ myeloid cells. The majority were Ly6C^+^ Mo/MØ, while a minority were Ly6G^+^ neutrophils (Fig. 3A,B, Supplemental Fig. 8). Amongst Ly6C^+^ Mo/MØs, the subset of Ly6C^lo^ cells have previously been shown to be important in normal thrombus clearance^33^. Importantly, lack of IL-6 did not alter Ly6C^lo^ cell frequency (Fig. 3C). Given that the Ly6C^+^ are most numerous and the likely source of IL-6, we performed adoptive transfer of labelled Td Tomato BMDM into WT and IL-6^−/−^ mice (Fig. 3D), and showed reversal of impaired VT resolution at 8 days post thrombosis (Fig. 3E; for comparison without adoptive transfer see Fig. 2F).

### IL-6 and inflammatory cells in human DVT

Given the chronicity of IL-6 elevation in the thrombus, we assessed human femoral vein chronic DVT from endophlebectomy specimens as previously described^47^. These specimens were from patients with > 6 month old femoral vein obstruction, who were treated surgically to improve venous inflow. Co-staining with gp130/CD126 showed marked staining, with modest macrophage CD68^+^ cellular co-localization (Fig. 3F). These data suggest Mo/MΦ have IL-6 responsiveness within the post thrombotic tissue, particularly around neovascular channels.

### Endogenous IL-6 promotes MMP9 expression and total MMP activity in BMDMs

**Figure 4:**
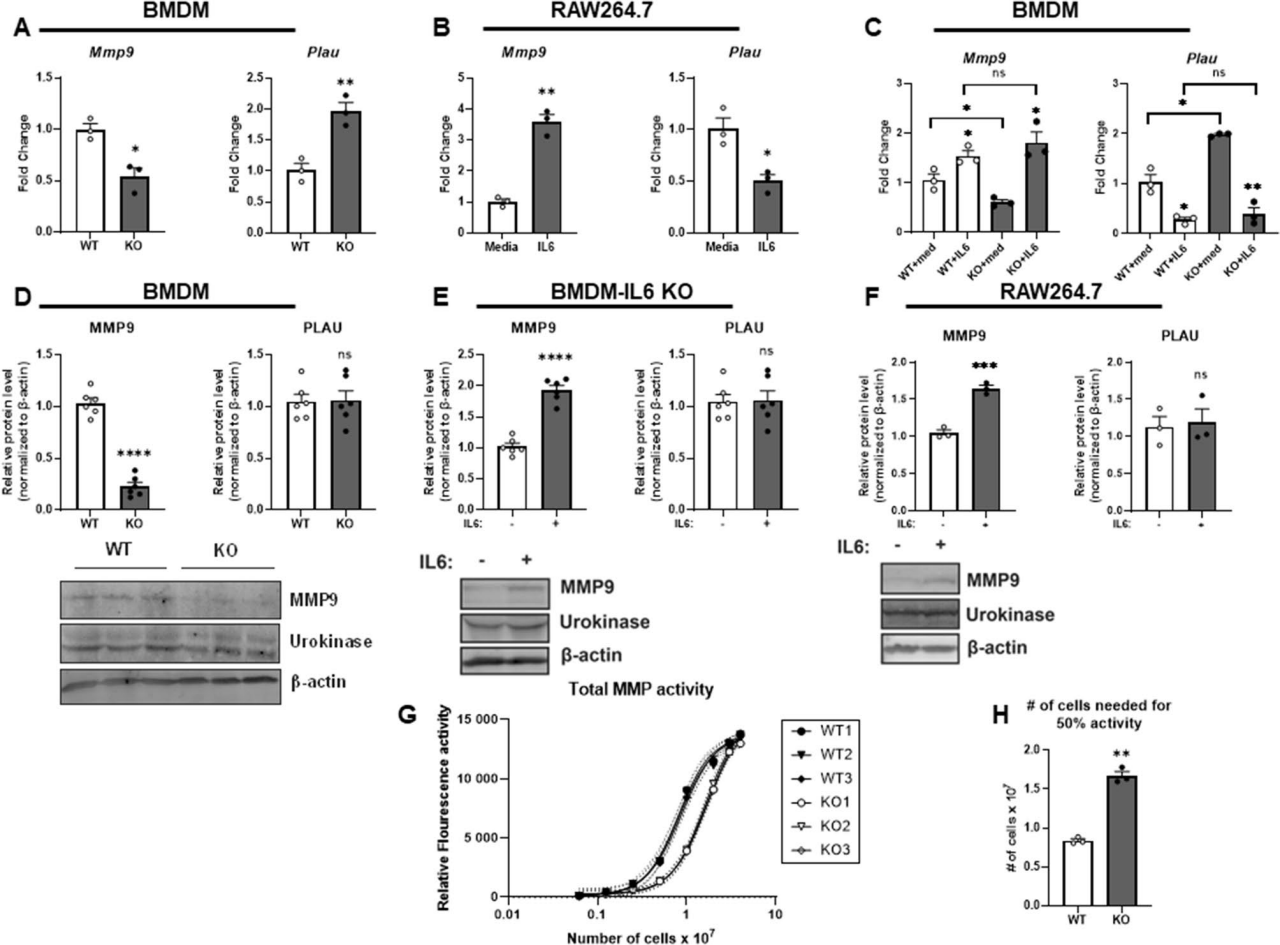
Endogenous IL6 promotes MMP9 expression and total MMP activity in BMDMs. (**A**, **B**) mRNA levels of the indicated factors were assayed in BMDMs derived from WT and I-L6^−/−^ mice and in RAW264.7 cells (**B**), respectively. (**C**) mRNA levels of *Mmp9* and *Plau* were assayed in the indicated BMDMs after 24 h after stimulation with recombinant murine IL6 (20 ng/mL final concentration, 24 h). Data representative of 3 independent experiments and 6 biologic replicates. (**D**) Protein levels of MMP9 and urokinase were analyzed using immunoblotting in BMDMs derived from WT and IL-6 deficient mice with quantification of MMP9 and urokinase protein levels relative to total protein loading. (**E**) Protein levels of MMP9 and Urokinase were assayed in the BMDMs and (**F**) RAW264.7 cells after IL-6 stimulation. Gel blots cropped from different parts of the gel are presented, with white line separation. Quantification of protein levels relative to total protein loading is shown in the upper panels. All western blots performed with n = 6 biologic replicates. (**G**) Total MMP activity was assayed in the indicated BMDMs and represented as measured MMP activity per concentration of cell lysate to produce the dose–response curves which are shown. Data shown is representative of n = 3 biologic replicates per group, performed with 3 technical replicates per cell lysate concentration. (**H**) Averaged calculated cell concentration required to achieve half maximal MMP activity per the interpolated dose response curves for the indicated BMDMs is shown. All data presented as mean ± standard error of the mean. Normally distributed data was compared utilizing unpaired student’s test with Welch’s correction. *n.s.* not significant, *p < 0.05, **p < 0.01, ***p < 0.001, ****p < 0.0001.

Given the impaired recanalization of resolving VTs in IL-6^−/−^ mice, we sought to identify differential expression of fibrinolytic, proteolytic, and angiogenic factors important in thrombus resolution among IL-6^−/−^ and WT monocytes. Analysis of mRNA transcripts from BMDMs derived from IL-6 deficient mice and WT controls showed a 47% decreased expression of *Mmp9* and twofold increased expression of *Plau* (urokinase) in IL-6 deficient BMDMs (Fig. 4A). Conversely, stimulation of immortalized murine macrophages (RAW264.7 cells) with recombinant murine IL-6 yielded a 3.6-fold increase in *Mmp9* expression and a 51% suppression of *Plau* expression, suggesting reciprocal regulation of *Mmp9* and *Plau* transcription by IL6 in these contexts (Fig. 4B), similar to our findings in vivo in mice with gene deletions of upa or *Mmp2*^48^. Unlike for *Mmp9*, the mRNA levels of *Mmp2* were unaffected by both IL-6 loss and recombinant IL-6 stimulation (Supplemental Fig. 9). The mRNA levels of vascular endothelial growth factor A (*Vegfa*) were unaffected by IL-6 loss but were induced in response to IL-6 stimulation (Supplemental Fig. 9). Similar to the results in RAW264.7 cells, stimulation of both WT and IL-6 knockout BMDMs yielded an increase in mRNA expression of *Mmp9* and suppression of *Plau* levels (Fig. 4C), suggesting that IL-6 deficient cells remained responsive to exogenous IL-6 stimulation. However, only *Mmp9* and not urokinase protein levels, were similarly suppressed in IL-6 deficient BMDMs compared to BMDMs isolated from WT mice (Fig. 4D). Furthermore, Mmp9, and not urokinase protein levels were increased in both BMDMs and RAW264.7 cells after IL-6 stimulation (Fig. 4E,F), thereby identifying Mmp9 as an inducible IL-6 dependent factor which may be important in the context of VT resolution. Analysis of total MMP activity in IL-6 deficient and WT BMDMs using a fluorescent substrate-based assay revealed impaired MMP activity in IL-6 deficient BMDMs, as seen by the rightward shift of the concentration-RFU curve (Fig. 4G) and an increased calculated cell concentration required to achieve half maximal MMP activity (Fig. 4H). These results suggest that IL-6 deficient BMDMs that generate Mo/MØ responsible for VT resolution are deficient in MMP9, and thus may contribute to overall decreased proteolytic capacity.

## Discussion

Venous thrombosis represents a major source of morbidity and mortality worldwide, and to date, no therapy outside of thrombolysis or anticoagulation has been proven to accelerate thrombus resolution^49^. From this series of experiments, we have characterized the role of IL-6 in two models of VT which replicate clinical venous thrombosis; namely, partial and complete blood stasis^50^. We demonstrate that IL-6 and IL-6 receptors for classical leukocyte are present in all thrombi and in the vein wall, are elevated locally in the setting of normal circulating IL-6 and exhibit a time-dependent increase following VT induction. The predominant mid-time point IL-6 positive cell type recruited to the thrombus are Ly6C^lo^ monocytes. Global IL-6 gene deletion resulted in larger collagen-rich thrombi with fewer neovascularization channels, despite a similar number of Ly6C^lo^ monocytes, and could be rescued by adoptive transfer of Ly6C^+^ IL-6^+/+^ monocytes. We further demonstrated that IL-6 stimulates monocyte-derived MMP9 expression, which has a defined role in thrombus resolution^26^. Lastly, the presence of CD126 + cells in human post-thrombotic tissue suggests a role in thrombus resolution, although at later time frame than experimentally analyzed.

Acute DVT is associated with elevations in pro-inflammatory cytokines such as IL-1β, IL-6, and C-reactive protein^51^. Interleukin-6 is a prototypical inflammatory cytokine, that has numerous cell specific effects via a cis and trans-signaling mechanism^13–15^. Nonvascular diseases associated with IL-6 include rheumatoid arthritis, systemic lupus, and osteoporosis^52^. More recently, acute COVID-19 infection has both elevated IL-6, and anti-IL-6 therapy is beneficial^53^. IL-6 has been shown to play a role in thrombogenesis and resolution^17,54^, but not as a key mediator of fibrinolysis^55^. Our findings that IL-6 gene deletion impaired thrombus resolution in a murine VT model are surprising, but supported by reports of delayed recovery of IVC blood flow in thrombosed IL6^−/−^ mice^54^. We found that IL6 gene deletion did not affect thrombus formation, contrary to data suggesting that anti-IL-6 inhibition can impair thrombogenesis^18^. These discrepancies may be due to timing of analysis, the models used such as stenosis as compared with stasis, and antibody inhibition as compared with global IL-6 genetic deletion.

Mo/MΦ are a multifunctional leukocytes involved in VT resolution^7,40,56,57^: they clear necrotic cells and matrix debris^58–61^, are a major source of fibrinolytic enzymes, and promote thrombus neovascularization^1,3,62^. They are classified by their inflammatory or anti-inflammatory functions^63^. We and others have previously shown that anti-inflammatory Ly6C^lo^ monocytes are essential to promoting VT resolution^33,64^. Within the myeloid cell cohort recruited to the resolving thrombus, Ly6C^lo^ monocytes were the predominant IL-6 positive cell, and with deletion, a proinflammatory Mo/MΦ phenotype dominated. Adoptive transfer of IL-6^+^ monocytes restored normal thrombus clearance, suggesting that these cells are a critical source of IL-6 in the mid-term resolving thrombus. These data augment our current understanding of the role of leukocytes in thrombus resolution by implicating monocyte-derived IL-6 as an essential mediator.

Human specimens confirmed IL-6 responsiveness in chronic DVT macrophages, highlighting the role of the Mo/MΦ as both IL-6 source and effector cell. IL-6 treatment of murine Mo/MΦ has been shown to increase gene expression of enzymes involved in thrombus digestion: uPA, MMP-2 and MMP-9^54^. Global genetic deletion of urokinase, MMP-2 and MMP-9 in our VT mouse models have confirmed their roles in mediating thrombus resolution^2,26^. We confirmed amongst a panel of angiogenic, proteolytic and fibrinolytic enzymes that in vitro IL-6 agonism of Mo/MΦ primarily stimulates MMP9 expression, production and activity. We also found as previously described that IL-6 stimulated urokinase expression^54^, but did not find significant changes in enzyme protein levels, suggesting that MMP9 is the primary proteolytic enzyme induced by IL-6 in Mo/MΦs.

A positive association between MMP9 and thrombus resolution has been previously reported in a stenosis model of VT^65,66^. Our study is the first to our knowledge to specifically study the temporal thrombus matrix outcomes in the setting of a suppressed IL-6-MMP-9 axis, with several provocative findings. First, we discovered that robust fibronectin deposition at mid time points preceded formation of a collagen-rich thrombus at late time points. Fibronectin composition is regulated by the presence of flow^67^, and proteolytic enzymes, primarily MMP-9^46,68^. Robust fibronectin deposition precedes collagen deposition in animal models of lung fibrosis and aberrant wound healing^42,43,69,70^. These data suggest that thrombus collagenous transformation occurs in a manner analogous to tissue fibrosis with relation to fibronectin content.

Secondly, we discovered impaired neovascularization in IL-6 deficient mice. Neovascularization likely contributes to thrombus resolution, in part by multi-functional monocytic cells^71^, with impaired neovascularization associated with larger VT^72,73^. Both fibronectin and MMP-9 have been shown to be important positive regulators of neovascularization in other tissue beds^74–77^. The fact that in our model IL-6 deficiency neovascularization was impaired despite high levels of fibronectin suggests that MMP9 may play a more predominant role in the post thrombotic setting. Additionally, conversion to a collagenous thrombus in IL-6 deficiency may be more resistant to innate fibrinolysis/proteolysis. Defective thrombus neovascularization and subsequent increased fibrosis has also been described as a pathogenic mechanism of pulmonary hypertension post PE, highlighting the need to understand these processes across the spectrum of venous thromboembolism disorders^78^.

Limitations are present in this study. Humans are the only mammal that has the propensity to develop spontaneous VTE. Therefore, any animal model will only partially recapitulate human VT, and have advantages and disadvantages^24^. The advantage to the partial and complete blood murine stasis models, as used in this study, are that both are reliable and well-established, simulate conditions that occur clinically as shown by duplex ultrasound (partially and totally occlusive thrombosis), and offer the ease of genetic manipulation to test molecules of interest^24^. Furthermore, chronic changes in the human post-thrombotic vein wall bear several parallels to changes seen in the experimental murine chronic VT^27,79^. The experiments herein suggested a model-determined effect of IL-6 actions. For example, IL-6^−/−^ was associated with a milder phenotype of impaired resolution in the stenosis model. It may be IL-6’s actions on fibrinolysis and neovascularization are more important in a totally static thrombus than one with some peri-thrombus blood flow. Certainly, other vein wall and thrombus responses such as NETS and endothelial-mesenchymal transformation are model dependent^80,81^. Given our previous published data on the essential role of Ly6C^lo^ monocytes in normal thrombus resolution, the high preponderance of IL-6^+^ staining in the intrathrombus Ly6C^lo^ cells and the restoration of thrombus resolution with adoptive transfer of IL-6^+^ monocytes, we assume that this subpopulation of IL-6^+^Ly6C^lo^ cells is likely the main driver of this phenotype. Additional future studies that would more definitively address the role of this cellular population would include: adoptive transfer of IL-6^+^Ly6C^lo^ cells into a post thrombotic IL-6 deficient mouse, (which was not feasible within the constraints of this model), and utilizing a transgenic IL-6^−/−^ monocyte specific knockout mouse.

In conclusion, our findings suggest that IL-6 signaling plays a significant role in thrombus resolution, dependent on the mechanism of VT as determined by the presence or absence of peri-thrombus blood flow. Our research suggests that the monocyte-IL-6-MMP9 axis represents a potential non-anticoagulant pharmacologic target that may aid thrombus resolution in patients with totally occlusive DVT. Others have shown exogenous IL-6 can accelerate VT resolution^54^. However, the translational challenge is to be able to locally target this proinflammatory cytokine to limit unwanted systemic proinflammatory cytokine effects. Future studies evaluating the specific role of MMP-9 in degrading thrombus fibronectin and on the role of fibronectin in subsequent thrombus collagen deposition may provide additional insight towards identifying non-anticoagulant therapies for residual obstructive DVT.

## Supplementary Information

Supplementary Figure 1.

Supplementary Figure 2.

Supplementary Figure 3.

Supplementary Figure 4.

Supplementary Figure 5.

Supplementary Figure 6.

Supplementary Figure 7.

Supplementary Figure 8.

Supplementary Figure 9.

Supplementary Table 1.

## Data Availability

The datasets used and analyzed during the current study available from the corresponding author on reasonable request (PK Henke, henke@umich.edu).
