# Supplementary figures and images for: Experimental venous thrombus resolution is driven by IL-6 mediated monocyte actions
Source: Sci Rep. 2023 Feb 24;13:3253. doi: 10.1038/s41598-023-30149-2 (PMC9951841; doi:10.1038/s41598-023-30149-2)

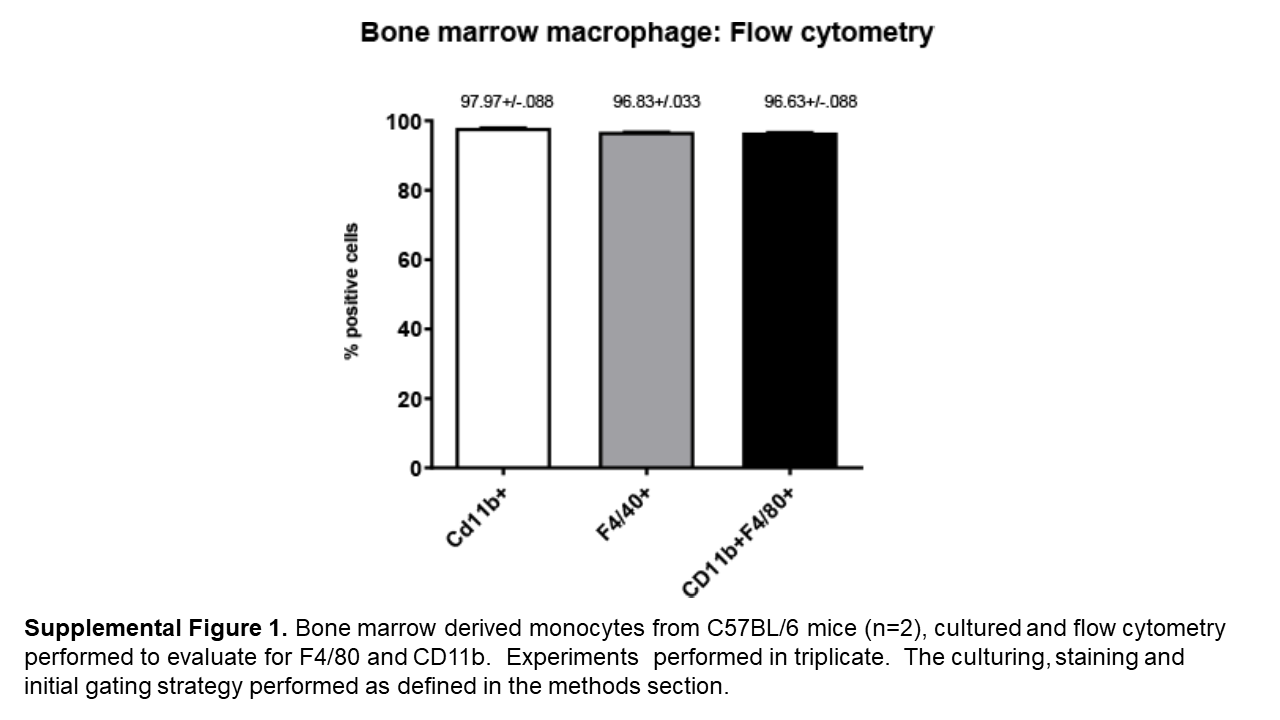

Supplement: Supplementary file 1 — Supplementary Figure 1. [file 41598_2023_30149_MOESM1_ESM.tif]

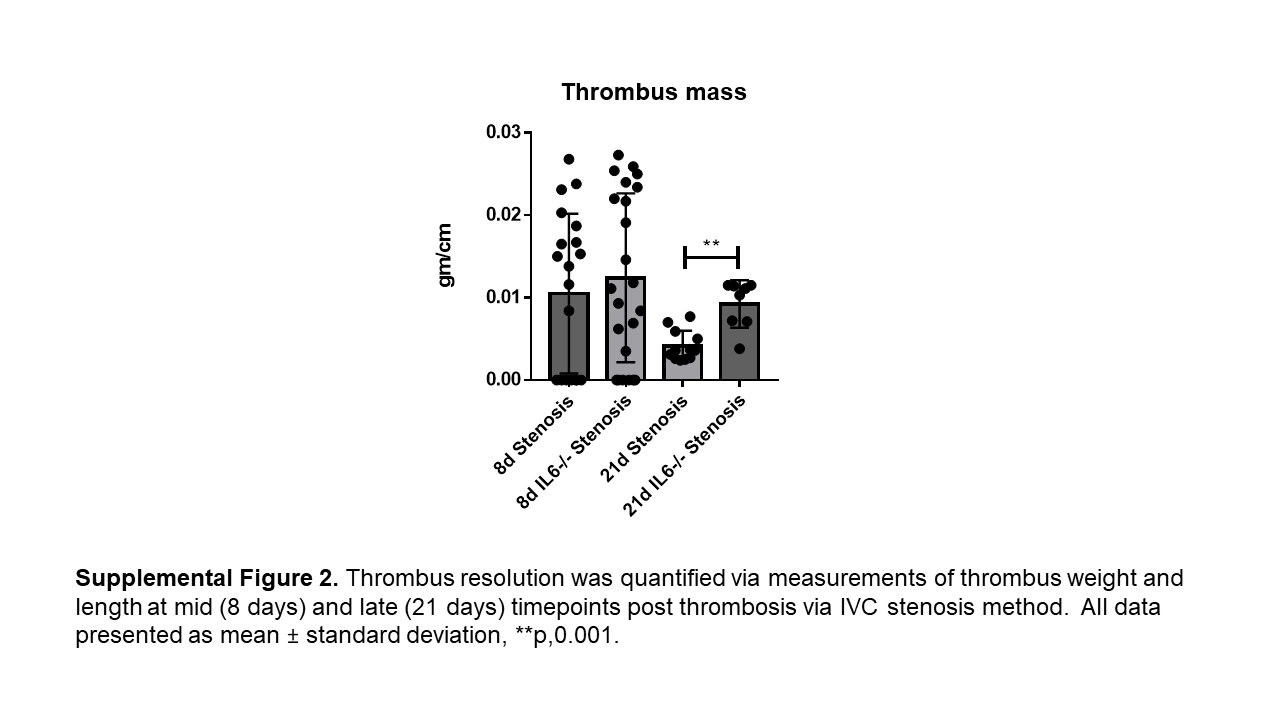

Supplement: Supplementary file 2 — Supplementary Figure 2. [file 41598_2023_30149_MOESM2_ESM.tif]

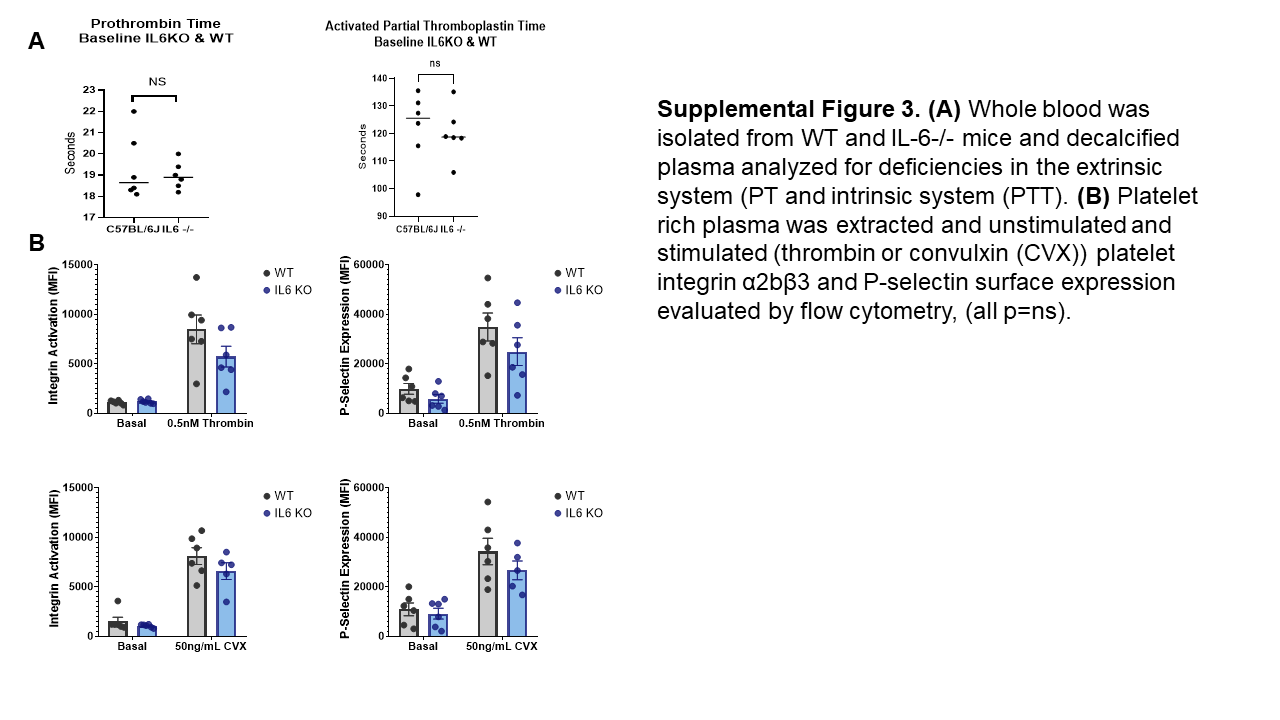

Supplement: Supplementary file 3 — Supplementary Figure 3. [file 41598_2023_30149_MOESM3_ESM.tif]

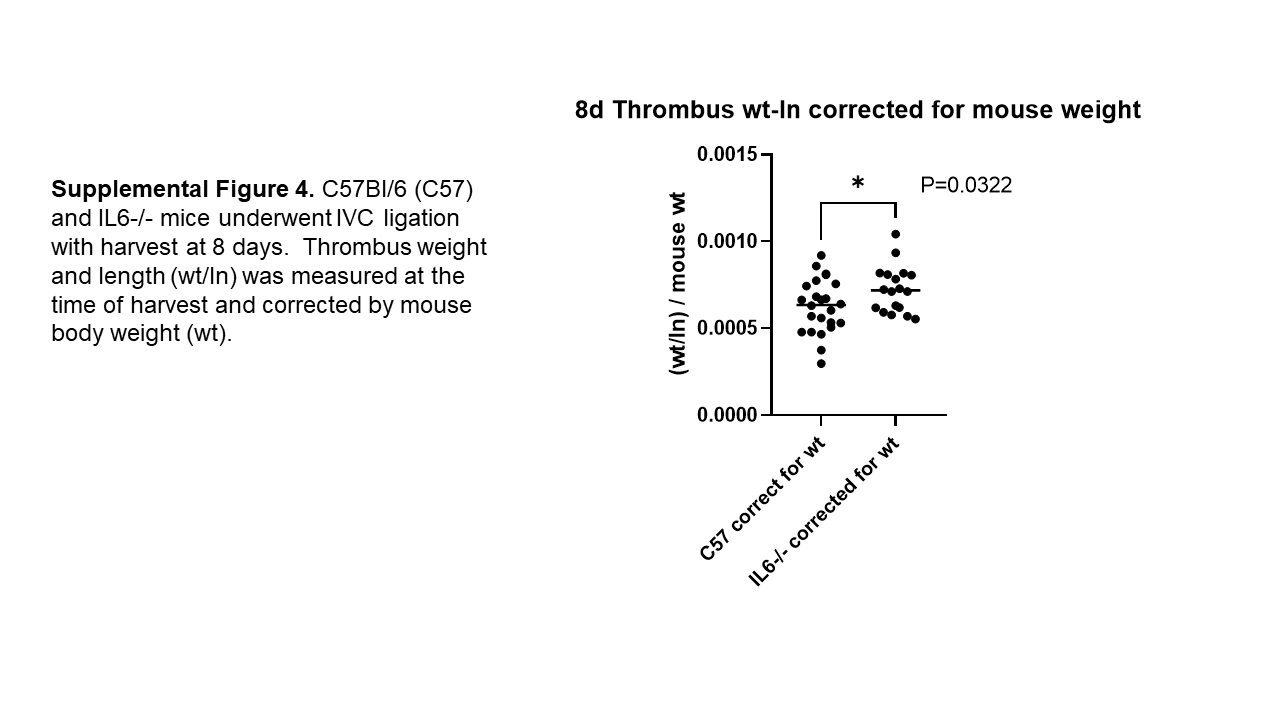

Supplement: Supplementary file 4 — Supplementary Figure 4. [file 41598_2023_30149_MOESM4_ESM.tif]

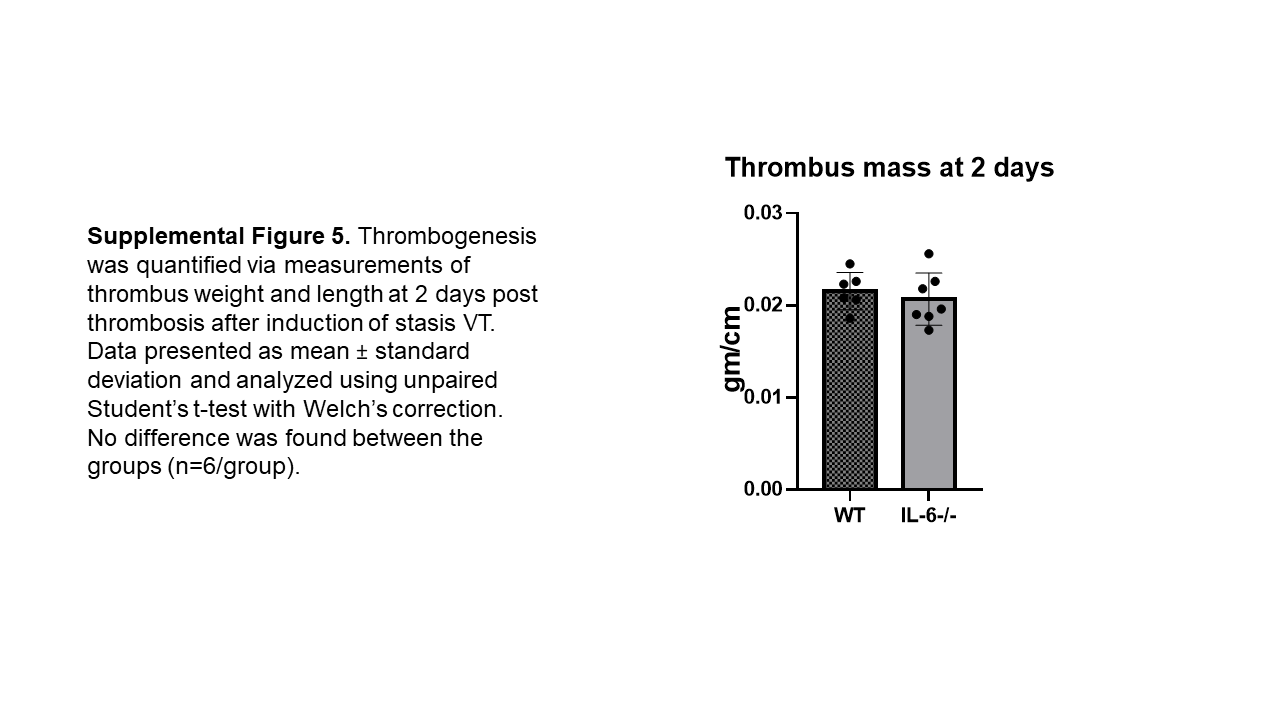

Supplement: Supplementary file 5 — Supplementary Figure 5. [file 41598_2023_30149_MOESM5_ESM.tif]

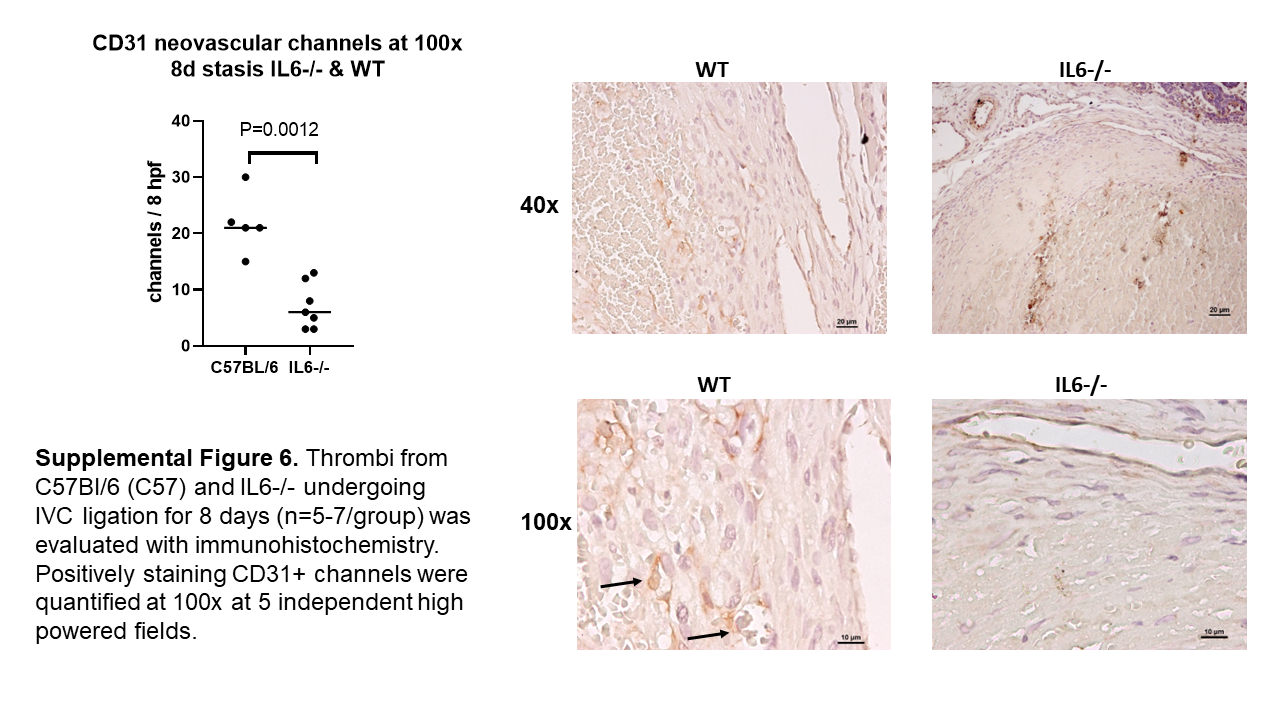

Supplement: Supplementary file 6 — Supplementary Figure 6. [file 41598_2023_30149_MOESM6_ESM.tif]

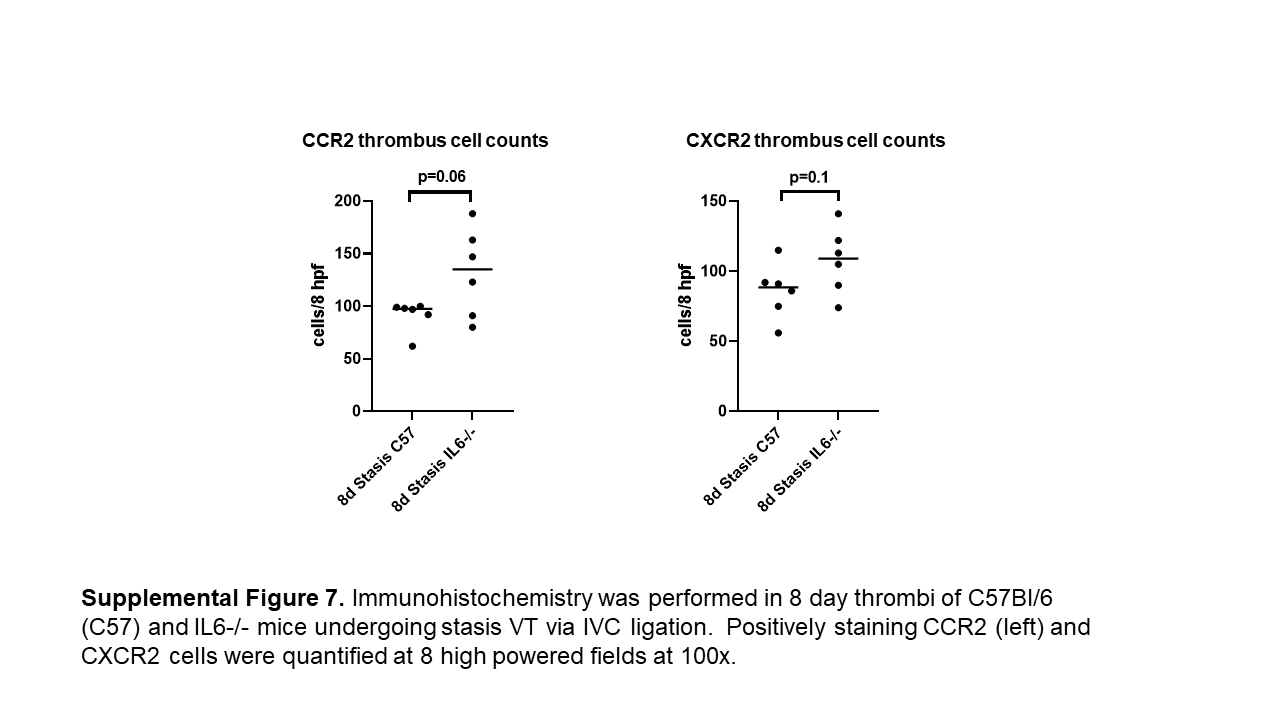

Supplement: Supplementary file 7 — Supplementary Figure 7. [file 41598_2023_30149_MOESM7_ESM.tif]

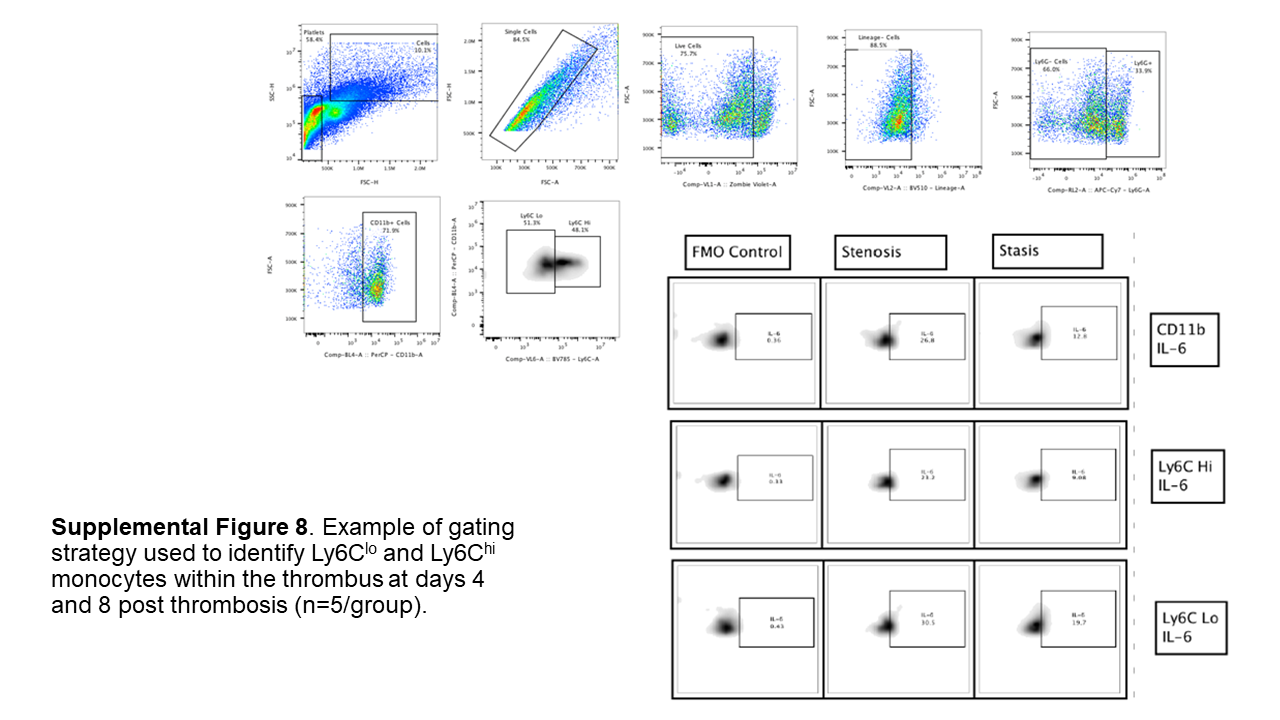

Supplement: Supplementary file 8 — Supplementary Figure 8. [file 41598_2023_30149_MOESM8_ESM.tif]

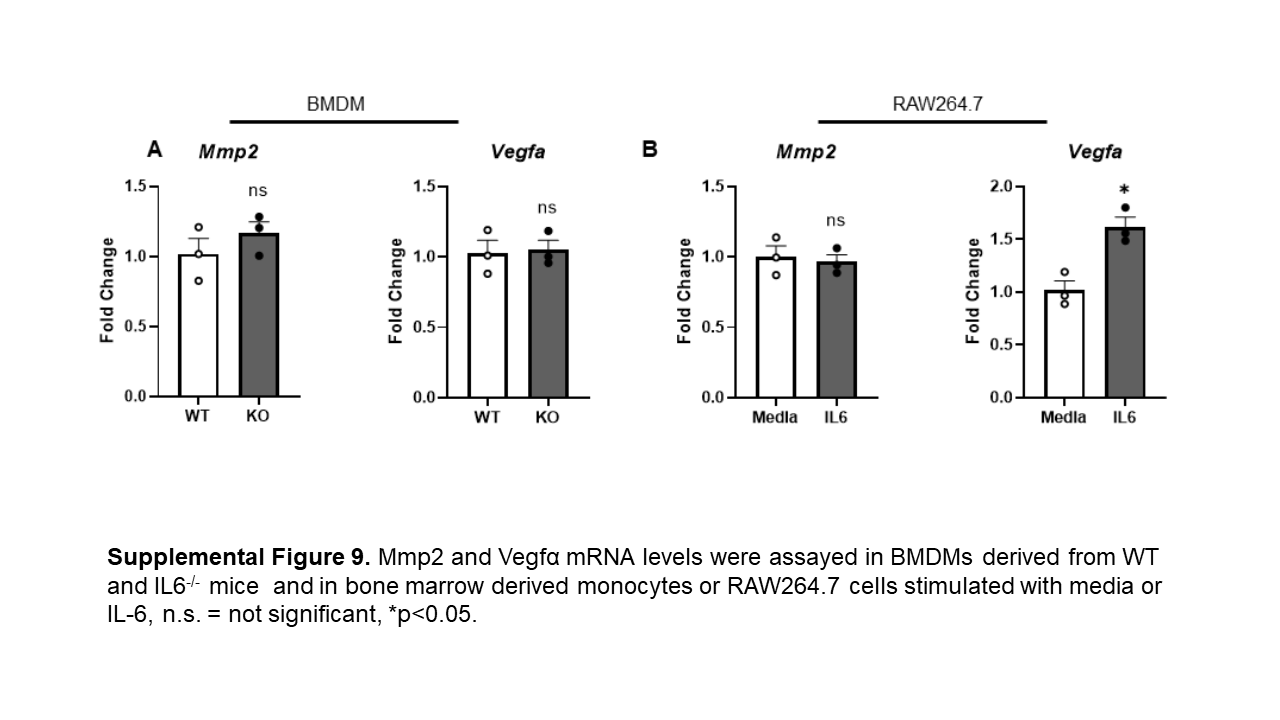

Supplement: Supplementary file 9 — Supplementary Figure 9. [file 41598_2023_30149_MOESM9_ESM.tif]
