# Supplementary material for: Experimental venous thrombus resolution is driven by IL-6 mediated monocyte actions
Source: Sci Rep. 2023 Feb 24;13:3253. doi: 10.1038/s41598-023-30149-2 (PMC9951841; doi:10.1038/s41598-023-30149-2)
Supplement: Supplementary file 10 — Supplementary Table 1. [file 41598_2023_30149_MOESM10_ESM.docx]

Supplemental Table 1:

Primer Sequences:

| **Gene** | **Accession Number** | **Sequence** | **Tm (_­­­_^o^C)** | **Amplicon Length (bp)** |
| --- | --- | --- | --- | --- |
| *Mmp2* | NM_008610.3 | F: 5’- GGACCCCGGTTTCCCTAAG-3’  R: 5’-CAGGTTATCAGGGATGGCATTC-3’ | 57.6  55.6 | 58 |
| *Mmp9* | NM_013599.5 | F: 5’- GCTGGACTCCGCCTTTGAG-3’  R: 5’- TTTGACGTCCAGAGAAGAAGAAAA-3’ | 58.5  54.4 | 62 |
| *Plau* | NM_008873.3 | F: 5'-TGTGAGATCACTGGCTTTGGAA-3'  R: 5'-GGACATTTTCAGGTTCCTTTGGATAG-3' | 56.5  54.0 | 74 |
| *Vegfa* | NM_001025250.3 | F: 5’- AAAGGCTTCAGTGTGGTCTGAGAG-3’  R: 5’- GGTTGGAACCGGCATCTTTATC-3’ | 58.1  56.2 | 184 |
| *Rpl38* | NM_001362918.1 | F: 5’-CGCCATGCCTCGGAAA-3’  R: 5’-CCGCCGGGCTGTCAG-3’ | 55.9  60.0 | 63 |
| *Eif3e* | NM_008388.2 | F: 5’-GGTTGGATGCCAAGATTGATTC-3’  R: 5’- GGGCGAGACTGCATTGTTG-3’ | 54.4  56.9 | 65 |
